# The Lived Experience of Lupus Flares: Features, Triggers, and Management in an Australian Female Cohort

**DOI:** 10.1155/2014/816729

**Published:** 2014-11-20

**Authors:** Marline L. Squance, Glenn E. M. Reeves, Howard Bridgman

**Affiliations:** ^1^Faculty of Health and Medicine, University of Newcastle, Callaghan, NSW 2308, Australia; ^2^Faculty of Science and Information Technology, University of Newcastle, Callaghan, NSW 2308, Australia; ^3^Autoimmune Resource and Research Centre, 2nd Floor, HAPS Building, John Hunter Hospital, New Lambton, NSW 2305, Australia; ^4^Hunter New England Health District, New Lambton, NSW 2305, Australia

## Abstract

Individuals living with lupus commonly experience daily backgrounds of symptoms managed to acceptable tolerance levels to prevent organ damage. Despite management, exacerbation periods (flares) still occur. Varied clinical presentations and unpredictable symptom exacerbation patterns provide management and assessment challenges. Patient perceptions of symptoms vary with perceived impact, lifestyles, available support, and self-management capacity. Therefore, to increase our understanding of lupus' health impacts and management, it was important to explore lupus flare characteristics from the patient viewpoint. Lupus flares in 101 Australian female patients were retrospectively explored with the use of a novel flare definition. Qualitative methods were used to explore patient-perceived flare symptoms, triggers, and management strategies adopted to alleviate symptom exacerbations. A mean of 29.9 flare days, with 6.8 discrete flares, was experienced. The study confirmed that patients perceive stress, infection, and UV light as flare triggers and identified new potential triggers of temperature and weather changes, work, and chemical exposure from home cleaning. The majority of flares were self-managed with patients making considered management choices without medical input. Barriers to seeking medical support included appointment timings and past negative experiences reflecting incongruence between clinician and patient views of symptom impact, assessment, and ultimately flare occurrence.

## 1. Introduction

Systemic lupus erythematosus (SLE, lupus) is a complex inflammatory illness with varied clinical presentations, providing management and assessment challenges to patients and practitioners [1, 2]. SLE patients live with daily background symptoms and only a few obtain symptom-free periods [3]. A life with lupus usually has an unpredictable pattern of symptom quiescence and periods of exacerbation commonly known as “flares.”

This paper explores lupus flares from the lived experience of 101 Australian female SLE patients. Data collection used a structured interview process. A novel flare definition, reflecting a chronic illness experience of symptom stability, punctuated by sustained exacerbation over a specified time period, was incorporated [4]. The paper documents findings of patient-perceived flare symptoms, triggers, and management strategies adopted to alleviate symptom exacerbations.

The study is part of the initial development phase of a wider cohort study exploring environmental determinants of SLE flare events, with data collected retrospectively over a whole year to incorporate potential seasonal influences upon patient wellbeing. Study goals were to

1. describe the patient experience of lupus flares, their features, and attributions of possible triggers and management;
2. compare these flare characteristics between patients meeting American College of Rheumatology (ACR) classification criteria [5] and those classified as having “borderline” lupus, falling within the undifferentiated connective tissue disease category as lupus-like disease.

Treating specialists often use validated tools (e.g., BILAG, SLEDAI, SELENA, and SLICC) [6–8] to monitor and objectively assess overall and organ-specific SLE activity and damage. However, despite recent consensus definition of lupus flare [9], differences between clinician and patient determination of flare events occur [10]. These arise when the definition of “flare” is based solely on biomarker measures and treatment changes, without sufficient recognition of an individuals' perception of quality of life (QOL) impact [1, 11–13]. Flare assessment is also complicated by (i) determination of timing; (ii) assumptions that clinician review occurs at each flare time-point; (iii) mistiming of diagnostic assessments; (iv) reluctance of patients to inform clinicians of symptom changes [14]; and (v) patient use of self-management strategies [15]. Severe flare events are often well supported by investigative confirmation, but determining milder flares with subtle symptom changes still proves difficult [7].

Presentation and definition of a lupus flare from a patient perspective is not readily explored. It is subject to the patient's perception of the illness impact as a reflection of their health values and priorities. Social support and capacity to self-manage, adopting necessary lifestyle changes, are also important considerations [12]. Symptoms reported by patients vary in personal importance. Therefore, exploring lupus flares from the patient's lived experience is an important research area that will increase understanding of lupus health impacts and treatment efficacy [13, 15–17].

## 2. Method

The study was a retrospective analysis of lupus flare characteristics within a large regional area of Australia. SLE diagnosis via the ACR criteria [5] was confirmed by health record audit. Participant demographic, lifestyle, and medical history was also collected via posted study specific questionnaires. Participant SLE management and flare history over a 12-month period were explored during a clinic appointment with the medical researcher where study questionnaires were collected and participants were interviewed via a structured interview process to document their perceptions of symptom activity (flare) and management over the previous 12 months. Data were of a self-reported qualitative nature.

Institutional review and approval according to the Declaration of Helsinki 2008 revision [18] were undertaken and received.

### 2.1. Study Population

SLE patients of the Autoimmune Resource and Research Centre (ARRC) and private immunology clinics in the Hunter/Central Coast regions of NSW, Australia, were invited to participate. Potential participants were drawn from clinic databases using disease codes “SLE” or “Lupus” as an identifier of potential study recruits. All identified patients were posted a letter of study invitation, along with study information and consent forms. Written consent was obtained for all aspects of the study.

Initial criteria included all identified SLE persons aged 18–80 years, but, due to low numbers of male respondents, the final study was limited to females. The cohort was predominantly of Caucasian ethnicity.

### 2.2. Health Record Audit

Health records of responding patients were reviewed to confirm SLE diagnosis and verify self-reported illness onset information, symptoms, disease history characteristics, and management plans, especially pharmaceutical usage. Evidence of a positive symptomatic improvement with a pharmaceutical challenge involving either a 3-month regime of hydroxychloroquine or 2 weeks of prednisolone was noted and used to identify a subgroup of “borderline” SLE patients.

Eligible participants met inclusion criteria and ACR SLE classification (SLE ACR 4+); a second comparator group was also included if 3 of the ACR criteria plus a positive pharmaceutical challenge were documented within the health record. The comparator group, borderline SLE ACR 3 + 1, falls within the ACR category for undifferentiated connective tissue disease.

### 2.3. Health Assessment and Interview

All participants attended a scheduled appointment to collect study questionnaires and also to document their lived experience of SLE symptom exacerbations, features, triggers, and management techniques, as well as perceptions of their flare frequency for the preceding 12 months. This flare assessment was undertaken via valid qualitative techniques with the use of a structured interview method [19].

Due to the retrospective nature of the study and the lack of standardized clinical assessment tools documented within health records, a novel subjective patient-centred flare definition was used. The definition was chosen because (i) it was easily understood; (ii) it contained reference to a chronic illness displaying periods of symptom stability, quiescence, and exacerbation; (iii) it recognised symptom background levels; and (iv) it best allowed exploration of the patient experience. Flare was defined as:

The appearance of a new clinical sign/symptom or the clinical worsening of a previous sign/symptom that had been stable for at least the previous 30 days and which persisted for a minimum of 24 hours [4].

The flare interview was conducted by the same medical researcher without clinician involvement. Each interview was in a verbatim structured format commencing with reading of the definition and a scripted clarification example. Fifteen questions exploring the patients' flare experience followed, without participant response prompting or discussion. All responses were concurrently documented by the researcher to allow free communication flow and to reduce recall bias. Participants confirmed accuracy of documented notes and comments at the end of the interview. Interview times ranged from 10 to 15 minutes in total. Structured interview format and question proforma used in the study have been provided as Supplementary information available online at http://dx.doi.org/10.1155/2014/816729.

Calculation of total counts of flare days and collection of data related to other perceived flare risk factors have been outlined in a previous paper [20].

### 2.4. Statistical Methods

Descriptive statistics summarised demographic information. Data were analysed with the use of inductive thematic analysis [21] to ensure complete data richness. Separate analysis was performed for “symptoms experienced in flares,” “identified triggers to flares,” and “management strategies for alleviating flare symptoms.”

To examine differences between assigned SLE groups, flare groups, and independent variables, continuous variables were analysed via one-way ANOVA, and categorical variables were analysed via Fisher's exact test of independence. All significant *P* values (≤0.05) were noted, with analysis performed using STATA v11.0 (StataCorp LP, College Station, Texas, USA).

## 3. Results 

A total of 159 patient health records were audited. Documented evidence of ≥4 out of 11 ACR criteria was confirmed in 83 participants, and 21 participants displayed ≥3 out of 11 ACR criteria plus a positive pharmacological challenge. Three participants reported “constant” flare and were excluded from further analysis. Demographic data for separate and combined groups of the remaining 101 participants are shown in Table 1.

Comparison of the separated groups showed no significant difference for most variables. Differences between groups were found for medical review periods with the “borderline SLE” reporting shorter periods. The “borderline SLE” group also had a larger proportion of participants not exceeding educational training past 15 years of age. Higher proportions of some ACR criteria were found in the “SLE” group: malar rash (71.3%, *P* = 0.01), photosensitivity (53.8%, *P* = 0.01), oral and nasal ulcers (36.3%, *P* = 0.00), and renal (46.3%, *P* = 0.03) and haematological disorders (48.8%, *P* = 0.02). Immune therapy medication (ITM) use was similar for each group; however, vitamin D supplementation was less in the “borderline SLE” group.

Combined participant group mean age was 48 (±12.9) years with mean disease duration of 7.7 (±6.7) years. Illness comorbidity was reported in 65.4%, with 61.4% of participants reporting more than one autoimmune illness. There was a high representation of participants reaching advanced or vocational level and above (57.5%) educational levels. A minority (18.75%) reported a SES of “below Australian average.”

Flare frequency information and reported symptoms along with relevant interview questions are presented in Table 2.

Twelve participants (15%) reported no flare events in the study period. All of these participants were in the “SLE group.” Self-reported individual flare events ranged from 0 to 52 flares (mean 6.8). Total flare days ranged from 0 to 240 days (mean 29.9 ± 38.8) with 2 participants reporting major adverse renal events resulting in prolonged hospitalization.

A majority of participants (63, 62.4%) reported a decreased flare frequency since implementation of medical management and a further 24 (23.8%) spontaneously created a 3rd response category, “remained the same.”

Participant perception of general health status since diagnosis was explored. Overall, general health improvement was recorded for 51 (50%) participants and deterioration was recorded for in 29 (28.4%). Despite having a nonflaring year, 3 (25%) of the 12 nonflaring participants still perceived their general health as deteriorating.

Current pharmacological immune therapy medications (ITM) for the cohort varied and included methotrexate, Plaquenil, prednisolone, Imuran, intravenous immunoglobulin, dapsone, and Cellcept. It is of note that 8 (67%) of the 12 nonflaring participants did not currently take steroidal based medications and, of these, 1 was not on any ITM. In comparison, 42 (47%) flare group participants did not take steroids and 15 (17%) were not on any ITM.

The broad spectrum of individual clinical presentation is reflected by the variety of symptom exacerbation descriptors reported. In total 376 symptom exacerbations were reported with a mean of 3.8 (±2.2: range 0–10) different symptoms for each participant. The 376 symptoms were thematically grouped into 29 separate codes with the 4 most frequent flare symptoms being joint and muscle pain (73.3%), fatigue (65.4%), skin rash (29.7%), and headaches (23.8%). Stress and anxiety, which are often linked as known flare triggers [3, 11, 22], were reported as flare symptoms along with depression and mood disturbance (3%). No significant difference between SLE groups was found for flare counts, frequency, general health VAS scores, or flare symptoms.

The unpredictable nature of symptom exacerbation and flares can impact on long term health and quality of life. This can lead to participants' awareness of subtle changes in symptoms and drawing links between flare triggers and changes in daily activities and agents. Within our cohort, 29 general trigger themes were identified from a total of 292 individual triggers. These are presented in Table 3.

A majority of participants (96%) identified triggers with a mean of 3 (±1.5, range: 0–6) different triggers identified by individual participants regardless of flare group. A small proportion of participants (4%) that reported flare events did not identify any trigger to these events.

The results were consistent with frequently cited triggers including stress (54, 53.5%), becoming run down (47, 37.6%), UV exposure (37, 36.6%), and infection (16, 15.8%), as well as triggers relating to physical responses to temperature, work, and sleep deprivation. Environmental influences on symptoms, in particular temperature sensations, included themes of “overheating” (20, 19.8%), “weather/changes in temperature” (20, 19.8%), and “cold temperature” (10, 9.9%). Interestingly, exposure to strong chemicals, specifically, bleach and pesticides were reported by 8 (7.9%).

Triggers associated with working and “being upset” are well-established associations in SLE which were also recorded within our cohort. The theme of “work” was represented by impact from workloads, deadlines, difficult bosses, hard jobs, and work hours (early and long). Physiological responses to changes in medications were reported in 17% of the nonflare group and only 6% of the flare group as an exacerbation-contributing factor.

To examine possible correlations between identified triggers, reported trigger themes and symptom exacerbations of the 89 flare group participants were cross-referenced in a matrix format, presented as Table 4. This matrix, whilst not documenting single reports of symptoms, does clearly show that flare symptoms of joint and/or muscle pain, fatigue, rash (malar, photosensitive and other), and headache were almost universally linked to the 15 most frequently reported trigger themes. The symptom of “swelling” (representing “*swollen finger*,” “*swollen hands*” or feet, “*swollen joints*,” and “*fluid retention*”) also featured as common cross-links to many triggers but was not linked to weather/sudden temperature changes or cold temperatures. Predictably, these 2 triggers along with “doing too much” and “infection” were cross-linked to symptoms in participants with known Raynaud's phenomenon comorbidity.

It is also of interest that 4 of the top 6 triggers identified in this study accord with established and known SLE flare triggers of stress, overexertion, UV exposure, and postinfective episodes. In addition to these our study also identified new potential environmental triggers to symptom exacerbation: sudden weather changes and overheating. This suggests that thermoregulatory dysfunction in people with SLE could have a role in disease activity and symptom changes.

The structured interview explored SLE management strategies employed with symptom exacerbations. The vast majority of participants nominated 1–5 strategies (mean 2.16 ± 1). Participant responses, tabulated within Table 5, show that 50% of participants change their steroidal medications to manage exacerbations, with 14% making these changes without medical advice. One participant, despite not taking steroids as part of her regular ITM, reported self-administration of steroids when flaring. This participant made the following comment:

* “I keep a supply just in case. I've been living with this for a long time and I know what works quickly and how to do it.”*

Participants did not readily notify their doctors of flare events. Only 25% notified their general practitioner and a lesser proportion (19%) notified their specialist; a further 14% clarified their response as “when I see them” and 6% “only if bad.” Participant comments noted different reasons for not accessing medical help, such as follows:

*“I can't get an appointment when I'm flaring, so I do all I can first, moving things around and hope it helps.” *

Comments also reflected a degree of resignation about their illness and the perception of a lack of empathy and understanding received from their medical supports:

*“I used to, but now they just look at you and say the numbers look fine, but I still feel like crap.”*

*“I only tell him when I see him, don't want to bother him. Sometimes the GP helps, but they don't understand really.”*

A total of 219 management strategies were reported and grouped into 12 separate themes. No differences were found between groups apart from the theme of “diet modification” for flare groups (*P* = 0.03), derived from small numbers of participants. Modifications specified concentrated on reducing processed foods, in particular breads or gluten containing foods, and increasing fresh fruits and vegetables. Four participants managed their flares by adopting a “bland” or “simple” diet due to “hot and spicy” foods being perceived as a flare trigger.

Changes in medications incorporating ITM as well as analgesics, anti-inflammatory medications, and topical ointments were employed in 67.3% of participants. Rest represented by “taking some time,” “time out,” “take it easy,” and “rest” was also reported in 66.3% of participants. One participant comment suggested the need to self-monitor and change behaviour:

*“I try to keep on top of things; I'm learning to be kinder to myself, saying no. It gets easier to know when it's happening; it's just a feeling I get to slow down. But it still frustrates me.”*

The strategy of pacing and planning rest periods was adopted by 11% of our cohort and was often reported in conjunction with relaxation (9%) and complementary therapy techniques including meditation. Overall complementary therapy usage was reported in 10% of participants. Therapies reported were a mix of exercise related activities, tai chi, Pilates, and yoga; homeopathic practitioner treatments (acupuncture, massage, and chiropractor); and a holistic soft tissue “touch” treatment (Bowen therapy) to relieve pain and promote healing. Interestingly, the use of herbs, probiotics, tonics, and homeopathy medications such as olive leaf, glucosamine, and fish oils, whilst reported as routine use in background questionnaires, was not reported as specific flare management strategies.

Changing daily behaviour including reducing workloads, avoiding computers, and changing environments were reported by 17% of participants. Of these, strategies to reduce the amount of outdoor hours and UV light, as well as staying within a warm or cool environment, were reported most frequently.

The use of simple everyday aids as a first-line treatment to alleviate symptoms was reported in 16% of participants as a management strategy. Aids specified included heating devices (wheat bags, gloves, heater, and electric blankets) and also hot or cold showers/baths with oils/minerals. Participants also reported increased usage of aids such as wheelchairs or walkers when flaring.

## 4. Discussion

This study was designed to improve our understanding of SLE flare symptoms, triggers, and management from the patient's viewpoint.

### 4.1. Flare Frequency

The majority of people living with SLE experience a daily symptom background level despite medical management. Heightened perception of symptom intensity can occur frequently; therefore the flare definition used specified that the variation needed to be sustained over a 24-hour minimum. Patient interpretation of flare events is based on a number of factors including overall illness severity, personal insight, and perceived degree of symptom impact [16]. Patients living with chronic illnesses use the word “flare” to explain multiple situations. Some patients report flare only when illness exacerbations are persistent, not explained by their activities, and require management help [13].

Eighty-eight percent of our cohort reported 1 or more flare events (mean 6.8); 2 participants reported weekly flare events; 56 (63%) of the flare group reported fewer than 5 flare events for the study year. Monthly flare events lasting for an average of 3.4 days were reported by 14 participants. Of interest, 11 of these were aged over 50 years and no longer experienced monthly menstrual cycles. In comparison, a prospective Norwegian study found an average monthly flare rate of 2.5 [23].

The specific timing and matching of symptoms to separate events could not be explored within this study. FitzGerald and Grossman [1] suggest that the assessment of flare timing and frequency is particularly difficult in observational cohorts with irregular follow-up: flares are variably coded as single or multiple events. Acknowledging this, flare frequencies reported in our study could reflect (i) a mixture of multiple single symptom events; (ii) a single continuous event with multiple different symptom exacerbations; or (iii) a single continuous event with the same symptom exacerbation resurfacing due to inadequate resolution.

Twelve participants reported no flares within the study period. Of these, 5 (42%) had a complex symptom spectrum with 6 or more ACR criteria. All nonflaring participants reported flare triggers and also management strategies used in the past to alleviate flare events. This indicates a level of illness understanding that allows optimisation of self-management strategies preventing and reducing flare events.

All except one nonflaring participant used nonsteroidal ITM medications for disease management. A small number commented that they had used steroids for management of exacerbations in the past, indicating that their pharmaceutical regime had altered over time.

### 4.2. Symptoms

Patient flare experiences differed in symptom presentation, triggers, and management strategies across individuals, but not between SLE groups. Cohort reported flare symptoms of joint and muscle pain, fatigue, rash, headaches, fevers, joint swelling, and cognitive clouding are consistent with other published literatures as being most frequently reported [3, 11, 24], although our rates did differ slightly, possibly indicating factors unique to the Australian environment. Particular differences included reduced reports of headache (23.8%) and cognitive impairment (11.9%) [17, 25]; also, joint and muscle pain (73.3%) was more frequent than fatigue (65.4%) [3, 11]. Gastrointestinal symptoms (13.9%) and shortness of breath (9.9%), not reported in other studies, were also frequent in this Australian cohort.

The flare behaviour similarities between “borderline” and “classic” lupus patients is noteworthy and is consistent with the proposal that lupus represents a spectrum of disease rather than a dichotomous state of lupus being present or absent.

Differentiation between symptoms of “normal” illness fluctuations, treatment side effects, and symptom exacerbations classified as “flares” can be difficult for both clinicians and patients [12]. Differences can lie in the level of importance placed on symptoms without sufficient regard to QOL impacts [1, 11–13]. This potential discrepancy between physician and patient flare definitions was demonstrated within this study by infrequent patient reports of some symptoms (ulcers, alopecia, seizures, and serositis) used within clinician assessment tools and ACR classification.

### 4.3. Triggers

The unpredictable nature of symptom fluctuations can impact greatly on an individual's QOL resulting in heightened awareness of symptom changes and recognition of suspected causal triggers [16, 24]. Confirming causal links is difficult and is not often undertaken; however, the role environmental triggers play in lupus development and flare has been described in the literature, with flare triggers of stress, overexertion, UV exposure, and infection being most often discussed [26–28]. Additional evidence also supports a role for oestrogens, drugs (e.g., hydralazine), and vitamin D as immune modulators in SLE [29, 30]. Our study supported established understandings of flare triggers with stress, overexertion, UV exposure, and postinfective episodes being most readily identified as potential triggers. The cohort identified these as the first, second, third, and sixth most frequent triggers along with several other perceived triggers.

The identification of weather changes (19.8%) and physiological responses to temperature changes such as “overheating” (19.8%) and “cold temperatures” (9.9%) are of great interest in light of predictions of climate change and weather instability. Dysfunction of the thermoregulatory system with either vascular or autonomic origins has been reported in SLE and other connective tissue disorders studies [31, 32]. Theories of causation vary and have included treatment/pharmacological driven responses, ischemia due to vasculitis [32], response to circulating complement-fixing autoantibodies directed against sympathetic and parasympathetic nervous structures [33], and proinflammatory cytokine activity [34]. However, more directed studies are required to investigate and establish lupus flare and temperature change associations.

The trigger-symptom matrix (Table 4) consistently linked frequently reported symptoms to reported flare triggers, independent of individual patient attributions. Not surprisingly, symptoms of fatigue and pain were cross-linked for all combinations examined including newly identified triggers associated with temperature alteration. It is of interest that potential associations between excessive proinflammatory cytokines have also been found for symptoms of fatigue, depression, and overheating in breast cancer patients [34] prompting thoughts of similar connection possibilities in people with SLE.

The matrix development was undertaken to aid in the identification and validation of trigger and symptom associations and was sensitive enough to successfully link a known symptom trigger (cold temperature) to Raynaud's symptoms in patients known to have this comorbid illness. This process could be further developed in larger multinational studies with reference to other comorbidities and also symptom spectrums of SLE patients allowing development of personalized self-management advice for day-to-day living.

Trigger discussions within published studies have also identified activities associated with work [20, 35, 36] and household chemicals [37–39] as being important considerations. These despite not being named within the top triggers were identified by our participants (work 8.9%, chemicals 7.9%) and will be the focus of future research.

### 4.4. Management

The majority of participants, independent of flare group, identified perceived triggers (95%) and adopted flare management strategies (98%). Coping skill development along with understanding symptom manifestations in many chronic illnesses is reported to increase patient resilience and self-efficacy [13]. This was also evident in our cohort with participants reporting that they seek out medical help once all self-management strategies have been exhausted. The reluctance of flaring participants, to both inform and involve treating clinicians, was based primarily on past experience and highlights the importance our participants placed on self-management. Concerns about costs, waiting times, travel distance, perceived lack of empathy, and understanding of symptom impact were expressed by our participants as in other studies [40–43].

Self-management strategies relied heavily on increasing rest periods, use of aids, and the addition of pain and anti-inflammatory medications prior to adjustment of ITM. Participants used retained steroid supplies and self-adjustment to alleviate symptoms and reduce need for medical interventions. This often occurred without adherence to staged dosage decline and without knowledge of medical risks. Exploration of published literature suggests that self-adjustment of medications is not unique to this study cohort [15, 44, 45].

Moore et al. [46] estimated that up to 49.8% of SLE patients have used alternative therapies. Usage was more common in patients of a younger age and higher level of education and those dissatisfied with medical care. Participants within our study often used alternative therapies, with increased usage during flares. Relaxation (8.9%) and the complementary therapies (9.9%) reported were a mixture of massage, Bowen therapy, acupuncture, and herbs. Flare attribution to a “build-up of toxins” was suggested by a few participants as a reason for flares events. Therapies were used as both a “detox” facilitator and symptom alleviator along with diet modifications such as adopting a fresh food or gluten-free diet.

Overall, participants reported that they did whatever they could to stay as well as possible. The fact that symptom flares still occurred despite all these ITM and lifestyle changes created distress through a constant reminder of disease. Over 62.4% of our participants reported a decreased flare frequency since medical management had commenced. Despite this reduction only 50% reported a general health improvement. This demonstrates that there is a considerable retained disease burden associated with living with lupus.

### 4.5. Strengths and Limitations

Retrospective studies have inherent limitations associated with participant recall bias; however they allow in-depth exploration of the lived lupus experience within a flexible and cost-effective study design. Consequently, they are useful in exploring hypotheses allowing refinement of focus for future research. Due to their flexibility the results can often be more generalized; however, results of our study should be taken in the context of a homogeneous female Caucasian population.

The study design incorporated an audit process to validate and standardise SLE classification and also allowed cross-correlation of self-reported medical information, therefore reducing potential recall and misclassification bias within methods.

Lupus flare was defined and viewed from a patient perspective. Individual participants may have varying interpretations of what constitutes a flare. To reduce participant misinterpretation, methods included a standardized structured interview inclusive of flare definition. The approach provided guidance to defining fluctuating symptoms as part of daily illness changes and sustained exacerbations more representative of flare events.

The use of a single structured interview without response exploration may have narrowed results, although participants freely volunteered comments and response clarifications. All relevant comments were included in the analysis. Importantly, this approach was supported by (i) convergent results found between this study and other SLE populations as well as other autoimmune illnesses and (ii) internal consistency using the trigger-symptom matrix.

## 5. Conclusion

This paper provides insight into the patient experience of SLE flare particularly perceived symptoms, triggers, and management strategies.

In addition to known flare triggers of stress, infection, and UV light, the study identified new and important potential flare triggers. The finding of patient associations between their flares and changes in temperature and weather is of particular concern in light of growing predictions of climate change. Triggers in relation to daily activities as part of working or home based cleaning (chemical exposure) raises potential for identifying specific triggers. Further research focusing on these activities may provide evidence for extension of current management guides for lupus and flare prevention.

In summary, we found that, each year, the average SLE patient suffers 30 days of symptom flares with around 7 discrete episodes, which are often self-managed without medical input. It was confirmed that lupus patients make considered choices about flare self-management. Many expressed barriers and reluctance to include medical support in their flare management, highlighting the need for improvements in patient/clinician interactions and the need for developing shared health management plans.

## Supplementary Material

The script outlines the interview process followed inclusive of the definition and explanatory example used prior to the set 15 questions. The script was designed to standardise interview processes and explore the experience of symptom exacerbations over the previous 12 months.

## Figures and Tables

**Table 1:** Demographic characteristics of patient participant groups.

	Borderline SLE ACR 3+1^*^ (*N* = 21)	SLE ACR 4+ (*N* = 80)	All patients (*N* = 101)	One-way ANVOVA

	Mean ± SD	Mean ± SD	Mean ± SD	*P* value

Age (years)	51.3 ± 9.9	47.7 ± 13.5	48.4 ± 12.9	
Diagnosis (years)	9 ± 8.3	7.7 ± 6.2	7.7 ± 6.7	
Medical review frequency	18.5 ± 14	21.3 ± 24.5	20.7 ± 22.7	0.01
Current health score (VAS scale)^**^	61.3 ± 15	55.3 ± 23.3	56.5 ± 21.9	0.02
Stress score (VAS scale)^***^	42.3 ± 29.8	50.1 ± 27.4	48.5 ± 28	
Outdoor hours per year	581.2 ± 428	490.5 ± 433	509.4 ± 431.5	
Average hours outdoors per day	1.6 ± 1.2	1.3 ± 1.2	1.4 ± 1.2	
Body Mass Index score	27.3 ± 5.9	27.4 ± 5.6	27.4 ± 5.6	

	no. (%)	no. (%)	no. (%)	*P* value

Comorbidity				
Autoimmune illness other than SLE	15 (71.4)	47 (58.8)	62 (61.4)	
Other illnesses not autoimmune	16 (76.2)	50 (62.5)	66 (65.4)	
Ethnicity				
Caucasian	21 (100)	78 (97.5)	99 (98)	
Other (Asian)		2 (2.5)	2 (2)	
Educational background				0.0
Year 9 (15 years)	9 (42.9)	8 (10.0)	17 (16.8)	
School certificate/leaving Certificate	4 (19.1)	24 (30.0)	28 (27.7)	
Higher school certificate	1 (4.8)	2 (2.5)	3.0 (3.0)	
Apprenticeship	1 (4.8)	4 (5.0)	5.0 (5.0)	
Tertiary (university/college)	3 (14.3)	36 (45.0)	39 (39.0)	
Postgraduate studies	2 (9.5)	6 (7.5)	8 (7.9)	
Socioeconomic status				
Above average	2 (9.5)	15 (18.8)	17 (16.8)	
Average	17 (80.9)	56 (70.0)	73 (72.3)	
Below average	2 (9.5)	9 (11.3)	11 (10.9)	
Smoking status				
Current smoker	2 (9.5)	6 (7.5)	8 (7.9)	
Past smoker	8 (38.1)	30 (37.5)	38 (37.6)	
Use immune therapy medications	17 (81)	67 (83.8)	84 (83.2)	
Use vitamin D supplementation	4 (19.1)	42 (52.5)	46 (45.5)	0.01
Use hormone supplementation	3 (14.3)	30 (37.5)	33 (32.8)	
Regular sun	12 (57.1)	39 (48.8)	51 (50.5)	
Regular sun + sunscreen	14 (66.7)	65 (81.2)	79 (78.2)	
Clinical ACR SLE features				
Malar rash	8 (38.1)	57 (71.3)	65 (64.4)	0.01
Discoid rash		3 (3.8)	3 (3)	
Photosensitivity	4 (19.1)	43 (53.8)	47 (46.5)	0.01
Oral/nasal ulcers		29 (36.3)	29 (28.7)	0.00
Arthritis	17 (81)	63 (78.8)	80 (79.2)	
Serositis	1 (4.8)	20 (25.0)	21 (20.8)	
Renal disorder	4 (19.1)	37 (46.3)	41 (40.6)	0.03
Neurological disorder	6 (28.6)	33 (41.3)	39 (38.6)	
Haematological disorder	4 (19.1)	39 (48.8)	43 (42.6)	0.02
Immunologic disorder	3 (14.3)	27 (33.8)	30 (29.7)	
Antinuclear antibody (ANA)	17 (81)	73 (91.3)	90 (89.1)	
Pharmacological challenge^****^	21 (100)	75 (93.8)	96 (95)	

^*^ACR 3+1 health record audit documented ACR criteria count + positive response to pharmacological challenge (^****^).

^**^Visual Analogue Scale score of 0–100: current Health (0) extremely Poor, (100) excellent.

^***^Visual Analogue Scale score of 0–100: stress level (0) no stress at all, (100) highly stressed.

^****^Documented positive response to pharmacological challenge of oral steroids (prednisolone) or hydroxychloroquine.

**Table 2:** Patient reported flare frequency and symptoms.

	Borderline SLE ACR 3+1 (*N* = 21)	SLE ACR 4+ (*N* = 80)	All patients (*N* = 101)

	Mean ± SD	Mean ± SD	Mean ± SD

Flare days calculated count	32.9 ± 38.7	29.2 ± 39.0	29.9 ± 38.8

	Number (%)	Number (%)	Number (%)

Flare day groups			
Any events	21 (100)	68 (85)	89 (88.1)
No events		12 (15)	12 (11.9)
<50 flare days	17 (81)	53 (66.3)	70 (69.3)
50–150 flare days	4 (19.1)	13 (16.3)	17 (16.8)
>150 flare days		2 (2.5)	2 (2)
Flare frequency since management^*^			
Decreased	9 (42.9)	54 (67.5)	63 (62.4)
Increased	4 (19.1)	10 (12.5)	14 (13.9)
Remained the same	8 (38.1)	16 (20)	24 (23.8)
General health since diagnosis^**^			
Remained the same	3 (13.6)	18 (22.2)	21 (20.6)
Improved	13 (59.1)	38 (46.9)	51 (50.0)
Deteriorated	5 (22.7)	24 (29.6)	29 (28.4)
Reported symptom exacerbation^***^			
Joint and muscle pain	18 (85.7)	56 (70)	74 (73.3)
Fatigue	12 (57.1)	54 (67.5)	66 (65.4)
Rash	5 (23.8)	25 (31.3)	30 (29.7)
Headaches	5 (23.8)	19 (23.8)	24 (23.8)
Fevers	4 (19.0)	11 (13.8)	15 (14.9)
Joint swelling	4 (19.1)	11 (13.8)	15 (14.9)
Abdomen/gastrointestinal	4 (19.1)	10 (12.5)	14 (13.9)
Brain fog/cognitive clouding	1 (4.8)	11 (13.8)	12 (11.9)
UV sensitivity	2 (9.5)	9 (11.3)	11 (10.9)
Shortness of breath	3 (14.3)	7 (8.8)	10 (9.9)
Raynaud's episodes increase	1 (4.8)	9 (11.3)	10 (9.9)
Infection	3 (14.3)	7 (8.8)	10 (9.9)
Chest pain	1 (4.8)	8 (10)	9 (8.9)
Skin dryness	3 (14.3)	6 (7.5)	9 (8.9)
Skin changes	3 (14.3)	5 (6.3)	8 (7.9)
Dizzy spells	3 (14.3)	5 (6.3)	8 (7.9)
Ulcers	1 (4.8)	6 (7.5)	7 (6.9)
Muscle weakness		7 (8.8)	7 (6.9)
Alopecia	1 (4.8)	5 (6.3)	6 (5.9)
Sleep disturbance	2 (9.5)	3 (3.8)	5 (5)
Neuralgia		4 (5)	4 (4)
Vision changes	1 (4.8)	3 (3.8)	4 (4)
Sore throat	3 (14.3)	1 (1.3)	4 (4)
Depression, anxiety, stress	1 (4.8)	2 (2.5)	3 (3)
Change of mood		3 (3.8)	3 (3)
Serositis		2 (2.5)	2 (2)
Anaemia		2 (2.5)	2 (2)
Seizures		2 (2.5)	2 (2)
Anorexia		2 (2.5)	2 (2)

^*^Has the number of events increased or decreased with ongoing management?

^**^In your opinion, since your diagnosis, has your general health improved, remained the same, or deteriorated?

^***^What symptoms have been exacerbated?

**Table 3:** Patient reported flare triggers.

	Borderline SLE ACR 3+1 (*N* = 21)	SLE ACR 4+ (*N* = 80)	All patients (*N* = 101)
	Number (%)	Number (%)	Number (%)
Could you identify any triggers to these events?			
Stress	13 (61.9)	41 (51.6)	54 (53.5)
Doing too much/run down	9 (42.9)	38 (47.5)	47 (37.6)
Sunlight exposure	5 (23.8)	32 (40)	37 (36.6)
Weather/temperature changes	4 (19)	16 (20)	20 (19.8)
Overheating	5 (23.8)	15 (18.8)	20 (19.8)
Infection	3 (14.3)	13 (16.3)	16 (15.8)
Lack of sleep/insomnia	2 (9.5)	11 (13.8)	13 (12.9)
Cold temperatures	1 (4.8)	9 (11.3)	10 (9.9)
Work	2 (9.5)	7 (8.8)	9 (8.9)
Being upset	3 (14.3)	6 (7.5)	9 (8.9)
Strong chemical exposure	4 (19.1)	4 (5)	8 (7.9)
Change in medicines	2 (9.5)	6 (7.5)	8 (7.9)
Fluorescent light exposure	1 (4.8)	5 (6.3)	6 (5.9)
Foods (spicy, bread)	3 (14.3)	3 (3.8)	6 (5.9)
Hormonal periods		5 (6.3)	5 (5)

Note: Other triggers reported in small numbers were dehydration, alcohol, long car journeys, inactivity, Christmas, gardening, allergic reaction, isolation, smoking, grandchildren, air conditioning, excitement, late night, and partying hard.

**Table 4:** Flare group reported trigger-symptom cross-link matrix.

Flare group *N* = 89 Trigger number (%)	Symptom number (%)									
Stress 51 (57)	Pain^*^ 42 (82)	Fatigue 42 (82)	Rash 18 (35)	H. ache^*^ 15 (29)	Ab/GI^*^ 12 (24)	Fever 9 (18)	Swelling 9 (18)	UV^*^ 9 (18)	Br. fog^*^ 9 (18)	Chest^*^ 7 (14)

Doing too much/run down 39 (44)	Pain 32 (82)	Fatigue 32 (82)	Rash 11 (28)	H. ache 10 (26)	Fever 10 (26)	Br. fog 7 (18)	Swelling 6 (15)	RP^*^ 4 (10)	UV 4 (10)	Ab/GI 2 (5)

Sunlight exposure 33 (37)	Pain 28 (85)	Fatigue 27 (82)	Rash 15 (46)	Swelling 12 (36)	H. ache 10 (30)	MusW^*^ 5 (15)	RP 4 (12)	UV 4 (12)	Br. fog 3 (9)	Ulcers 3 (9)

Weather/temperature changes 20 (22)	Fatigue 15 (75)	Pain 12 (60)	Rash 6 (30)	Fever 6 (30)	RP 5 (25)	SOB^*^ 4 (20)	H. ache 3 (15)	Inf.^*^ 3 (15)	Chest 3 (15)	Skin^*^ 3 (15)

Overheating 18 (20)	Fatigue 14 (78)	Pain 12 (67)	Rash 7 (39)	SOB 5 (28)	H. ache 4 (22)	Br. fog 4 (22)	Swelling 4 (22)	Ab/GI 3 (17)	Skin, sleep^*^, fever, ulcers, vision^*^ 2 (11)	

Infection 14 (14)	Pain 12 (86)	Fatigue 12 (86)	Swelling 5 (36)	Br. fog 4 (29)	H. ache 4 (29)	MusW 4 (29)	RP 3 (21)	Inf. 3 (21)	SOB, Ab/GI, ulcers, alopecia, vision 2 (14)	

Lack of sleep/insomnia 13 (15)	Fatigue 13 (100)	Pain 11 (85)	H. ache 6 (46)	Rash 5 (38)	Fever 5 (38)	Swelling 3 (23)	Br. fog 3 (23)	Ab/GI 3 (23)	Chest 3 (23)	Neuralgia^*^ 3 (23)

Cold temperatures 10 (11)	Fatigue 9 (90)	Pain 6 (60)	RP 5 (50)	Fever 3 (30)	Dizziness 3 (30)	Rash, inf., chest, mood, MusW 2 (20)				

Work 7 (8)	Pain 5 (71)	Fatigue 5 (71)	Rash 5 (71)	UV 4 (57)	Br. fog, H. ache, Ab/GI, dryness, RP 2 (29)					

Being upset 8 (9)	Pain 8 (100)	Fatigue 7 (88)	Throat 3 (38)	SOB, fever, chest 2 (25)						

Strong chemical exposure 8 (9)	Pain 6 (75)	Fatigue 6 (75)	Rash 4 (50)	H. ache 3 (38)	Swelling 3 (38)	Dryness 3 (38)	SOB, skin 2 (25)			

Change in medicines 6 (67)	Pain 5 (83)	Fatigue, sleep, rash, fever 2 (33)								

Fluorescent light exposure 6 (67)	Pain 4 (66)	Fatigue 4 (66)	H. ache 3 (50)	Swelling 3 (50)	Rash 3 (50)	UV 3 (50)	Dryness, ulcer, Br. fog 2 (33)			

Foods (spicy, bread) 5 (56)	Pain 5 (100)	Fatigue 4 (80)	Ab/GI 3 (60)	H. ache, swelling 2 (40)						

Hormonal periods 5 (56)	Pain 5 (100)	H. ache 3 (60)	Skin 3 (60)	Fatigue, rash, Ab/GI 2 (40)						

^*^Pain: joint and muscle pain, H. ache: headache, Ab/GI: abdominal/GI symptoms, Br. fog: brain fog/cognitive clouding, chest: chest pain, UV: UV sensitivity, RP: Raynaud's phenomenon, MusW: muscle weakness, SOB: shortness of breath, Inf.: infection, skin: skin changes, sleep: sleep disturbance, vision: vision changes, and neuralgia: feeling of ant's crawling, pins, and needles.

**Table 5:** Flare management.

	Borderline SLE ACR 3+1 (*N* = 21)	SLE ACR 4+ (*N* = 80)	All patients (*N* = 101)
	Number (%)	Number (%)	Number (%)

Did you change your steroid levels?	12 (57.1)	38 (47.5)	50 (49.50)
Steroid adjustment by medical specialist	8 (38.1)	28 (35)	36 (35.6)
Steroid adjustment by self-management	2 (9.5)	12 (15)	14 (13.9)
P notification	7 (33.3)	18 (22.5)	25 (24.8)
“Only if bad”	2 (9.5)	3 (3.8)	5 (5)
Specialist notification	2 (9.5)	17 (21.3)	19 (18.8)
“Only if bad”		7 (8.8)	7 (6.9)
“When I see them”	2 (9.5)	13 (16.3)	15 (14.9)
Patient reported flare management method^*^			
Medication change (not specified)	11 (52.4)	57 (71.3)	68 (67.3)
Rest	13 (61.9)	54 (67.5)	67 (66.3)
Change behaviour	5 (23.8)	12 (15)	17 (16.8)
Use of aids (heat packs, wheelchair, walker)	2 (9.5)	14 (17.5)	16 (15.8)
Pace planning	2 (9.5)	9 (11.3)	11 (10.9)
Complementary therapies	2 (9.5)	8 (10.0)	10 (9.9)
Relaxation	1 (4.8)	8 (10.0)	9 (8.9)
Diet modification	4 (19.1)	3 (3.8)	7 (6.9)
Exercise	3 (14.1)	2(2.5)	5 (5)
Go to the doctor	1 (4.8)	4 (5)	5 (5)
Ignore	1 (4.8)	2 (2.5)	3 (3)
Hobby distraction from symptom		1 (1.3)	1 (1)

^*^How did you manage this/these event(s)?
