# Supplementary material for: The Lived Experience of Lupus Flares: Features, Triggers, and Management in an Australian Female Cohort
Source: Int J Chronic Dis. 2014 Nov 20;2014:816729. doi: 10.1155/2014/816729 (PMC4590935; doi:10.1155/2014/816729)
Supplement: Supplementary file 1 — The script outlines the interview process followed inclusive of the definition and explanatory example used prior to the set 15 questions. The script was designed to standardise interview processes and explore the experience of symptom exacerbations over the previous 12 months. [file 816729.f1.doc]

***Environmental Determinants of Lupus Flare Study***

**Flare Interview Script**

Thank you for taking the time to be part of this study into Lupus flares.

The following interview will explore your experience over the previous 12 months with exacerbations of your Lupus symptoms. The interview should take approximately 15 minutes of your time.

I ask that you answers the following 15 questions based on your own experience over the previous 12 months only. I will pause after each question for 10 sec to allow you time to gather your thoughts. However, I will not interrupt your responses or prompt your answers.

If you don’t have a response to a question asked please say so and we will move to the next question.

All you responses will be documented on the Flare checklist sheet and at the end of the interview you will be asked if the documentation is a correct representation of your responses.

At the start of the interview I will give you a definition of exacerbation or flare which I ask that you keep in mind when responding to questions.

After the definition I will give you an example of a symptom exacerbation or flare and then begin the 15 questions.

Do you have any questions?

**Flare checklist sheet** Participant Code:  **_ _ _ _ _ _ _ _ _ _**

**Date: / / Time commenced: Time finished:**

Lets begin.

An exacerbation or flare is defined as:

The appearance of a new clinical Sign / Symptom or the clinical worsening of a previous Sign / Symptoms that had been stable for at least the previous 30 days and which persisted for a minimum of 24 hours.

Poser CM et al. *Ann Neurol* 1983 :13(3); 227-231

This means that if as part of your illness you have a pain in your toe and everyday you wake up and your toe pain on a scale of 1-10 with 1 being minimal pain and 10 being the most pain possible, is at a level of 3 and has been at that level for the previous 30days or month, then this is sadly part of your illness symptoms and not considered to be an exacerbation or flare. However, if you wake up and your toe pain is at a level of 5 or more and this pain remains with you for more than 24 hours then for this study we will call this a symptom exacerbation.

Do you understand?

Based on the definition

**Flare checklist sheet** Participant Code:  **_ _ _ _ _ _ _ _ _ _**

**Date: / /**

Over the past 12 months have you experienced an exacerbation of your usual symptoms? Yes No

If yes, what symptoms have been exacerbated?

_________________________________________________________________________________________________________________________________________________________________________________________________________________________________________________________________________________

What was the approximate date of your last symptom exacerbation? ____ / _______

How often over the past 12 months has an exacerbation occurred? No. _______________

How long did this/ these event(s) last? Days _______________

___________________________________________________________

___________________________________________________________

___________________________________________________________

___________________________________________________________

Group Assignment

***1***

***2***

***3***

Could you identify any trigger to this/these events?

___________________________________________________________

___________________________________________________________

___________________________________________________________

___________________________________________________________

How did you manage this/these events?

___________________________________________________________

___________________________________________________________

___________________________________________________________

___________________________________________________________

Did you change your Steroid levels? Not on any Yes No

If yes, did you adjust the level by self-management or by medical advice? Self Medical Other _________________________

Did you notify your General Practitioner of this/these events? Yes No

Did you notify your Specialist of this/these events? Yes No

Has the number of events increased or decreased with ongoing management? Increased Decreased

Do you regularly see a medical professional for review for your illness management? Yes No

If yes, how often do see a medical professional? _____________ months

In your opinion, since your diagnosis has your general health:

improved remained the same deteriorated

**Thank you for your time**.

Do you think I have written down all your answers correctly? Yes No

Is there anything else you would like to add? Yes No

**Flare checklist sheet** Participant Code:  **_ _ _ _ _ _ _ _ _ _**

**Date: / /**

Additional information

------------------------------------------------------------------------------------------------------------------------------------------------------------------------------------------------------------------------------------------------------------------------------------------------------------------------------------------------------------------------------------------------------------------------------------------------------------------------------------------------------------------------------------------------------------------------------------------------------------------------------------------------------------------------------------------------------------------------------------------------------------------------------------------------------------------------------------------------------
